# Perceived Stress, Perceived Social Support, and Global Health in Adults with Chronic Pain

**DOI:** 10.1007/s12529-023-10250-6

**Published:** 2023-12-21

**Authors:** Elena Castarlenas, Santiago Galán, Ester Solé, Rubén Roy, Elisabet Sánchez-Rodríguez, Mark P. Jensen, Jordi Miró

**Affiliations:** 1 Unit for the Study and Treatment of Pain – ALGOS, Research Center for Behavior Assessment, Department of Psychology, Universitat Rovira I Virgili, Catalonia, Spain; 2 Institut d’Investigació Sanitària Pere Virgili, Universitat Rovira i Virgili, Catalonia, Spain; 3 Department of Rehabilitation Medicine, University of Washington, Seattle, WA USA

**Keywords:** Chronic pain, Social support, Perceived stress, Global physical health, Global mental health

## Abstract

**Background:**

Chronic pain is a common problem in adults that can have a significant impact on individuals’ quality of life and on society. The complex pain experience emerges from a dynamic combination of biological, psychological, and social factors. Previous research has shown that social support has positive effects on health-related outcomes through two mechanisms: direct-effects and stress-buffering effects. The aim of this study was to investigate the role that perceived stress, perceived social support, and their interaction play as predictors of global physical health and global mental health in adults with chronic pain.

**Method:**

One hundred sixty-five adults with chronic pain completed measures of pain, perceived stress, perceived social support, global physical health, and global mental health.

**Results:**

Perceived stress but not perceived social support made a significant and independent contribution to the prediction of global physical health; both perceived stress and perceived social support made independent contributions to the prediction of global mental health. The perceived stress × perceived social support interaction did not make a significant contribution to the prediction of either criterion variable. The results suggested that perceived stress has an impact on both global physical and mental health, whereas perceived social support associated mostly with global mental health. In addition, perceived social support does not appear to moderate the impact of stress on global physical and mental health.

**Conclusion:**

The findings are more consistent with a direct-effects model than a stress-buffering model of social support.

## Introduction

Chronic pain, defined as pain that persists for longer than 3 months, is a significant healthcare problem that remains one of the most prominent causes of disability worldwide [1]. Epidemiological studies, conducted in adults of different regions, estimate chronic pain prevalence to generally range from 10 to 30%, depending on how chronic pain is defined [2–5]. In addition to being a common problem, living with persistent pain is often a distressing experience that can have negative impacts on people’s physical and psychological health [6], seriously affecting the patients’ daily activities and quality of life [7].

Chronic pain is best viewed from a biopsychosocial perspective, which hypothesizes the experience of pain and its negative impact on physical and mental health is influenced by the dynamic interplay between a biological, psychological, and social factors [8–10]. Stress and social support are two key psychological factors in the biopsychosocial framework that have been shown to be related to health-related outcomes in individuals with chronic pain [11–13]. For example, stress has been found to be significantly and positively associated with pain severity and pain-related disability [14–16], while social support has been found to be associated with better physical and mental health in individuals with chronic pain [17–20]. In addition, evidence indicates that social support is a protective factor against the negative effects of pain on mental and physical health [21, 22].

Although the influence of social support and stress on adjustment to chronic illness has been widely studied, the mechanism by which both factors influence health-related status deserves further attention. Traditionally, two models have been proposed to explain the role of social support in adjustment to stress [23, 24]: a direct-effects model and a stress-buffering model. The direct-effects model hypothesizes that social support contributes directly to physical and psychological health across all levels of stress. The stress-buffering model hypothesizes that the negative impact of stress on health-related outcomes is buffered by the presence of social support; that is, social support has larger benefits for those experience higher levels of stress than for those experiencing lower levels of stress.

The stress-buffering hypothesis of social support has been examined in a large range of studies with non-clinical populations as well as in samples with a variety of health conditions. For example, in studies conducted with university students, social support has been found to buffer the negative impact of stress on mental health [25, 26] including depression [27, 28]. In studies with patients with various health conditions, the findings have mostly supported the buffering effects of social support on the association between stress and *health-related* status and outcomes. For example, evidence for this model has been found in studies with adults with type 2 diabetes [29], women survivors of gynecologic cancer [30], and with individuals with early-stage dementia [31]. However, Kornblith and colleagues [32] found that stressful life events and social support significantly and directly predicted participants’ psychological status across all levels of stress, consistent with the direct-effects but not with the stress-buffering model.

Both the direct-effects and the stress-buffering models have been examined in samples of patients with chronic pain. On the one hand, findings supporting the direct-effects model but inconsistent with the stress-buffering model have been reported in longitudinal [33, 34] and cross-sectional studies [35, 36] conducted in samples of individuals with rheumatoid arthritis. In all cases, social support did not moderate the association of stress on depressive symptoms [33, 34]. On the other hand, support for the stress-buffering hypothesis of social support has been found in studies with patients with rheumatoid arthritis when the criterion variable was depressive symptom severity [37] and psychosocial adjustment [38, 39].

As summarized above, mixed results have been found when testing the direct-effects and the stress-buffering models of social support on health outcomes. The inconsistency in the findings could be attributed, at least in part, to the methodological differences between the studies, particularly in the way stress is defined and assessed. For example, “stress” has been operationalized as the level of functional ability [34], the presence of arthritis [37], the level of pain intensity during the past week [36], and the level of disease activity and functional disability scores [38]. This inconsistency in how stress is operationalized makes difficult to compare results across studies.

Considering the extant evidence, additional research is needed to help determine whether social support buffers the effects of stress on mental and physical health and health-related outcomes in adults with chronic pain using specific measures developed to measure both social support and stress. The knowledge concerning these effects is important to help determine whether, and for whom, social support is a viable treatment target, as a way to help adults better manage chronic pain and its impact on their lives.

Given these considerations, the aim of this study was to increase the understanding of the role that perceived social support and perceived stress play in the prediction of global physical and global mental health (i.e., people’ general perception of their physical and mental health) in adults with chronic pain, by testing hypotheses emerging from the direct-effects and stress-buffering models of social support, with the purpose of examining which model is supported in a sample of adults with chronic pain. Consistent with the direct-effects model, we hypothesized that both social support and stress as perceived by the participants (thus, in this study, we use the terms “perceived social support” and “perceived stress,” respectively) would contribute independent variance to the prediction of global physical and global mental health in a sample of adults with chronic pain. In addition, in order to evaluate the stress-buffering model, we hypothesized that perceived social support would moderate the associations between perceived stress and global physical and global mental health, such that participants reporting more perceived social support would evidence weaker associations between perceived stress and global physical and mental health than participants reporting less perceived social support.

## Method

### Participants

Participants in this study were a sample of adults with chronic pain. Criteria for eligibility included (1) having a chronic pain problem (i.e., a pain complaint that had persisted or progressed for 3 or more months of duration), (2) being at least 18 years of age, (3) having a good comprehension of Spanish language (because the measures used were written in that language), and (4) having an electronic device (e.g., computer, smartphone, tablet) in order to access to the online survey.

The study enrolled 165 adults with chronic pain, of whom 153 (93%) were women. Participants’ age ranged from 18 to 68 years, and the average age was 43.68 years (SD = 9.81). One hundred sixteen (70%) of the participants reported that their pain was continuous, and 49 (30%) reported that it was recurrent. The most commonly reported chronic pain location was the area around the low back, lumbar spine, sacrum, and coccyx (*N* = 60; 37% of the participants). Other common pain sites were the upper shoulder and limbs (*N* = 30, 18%) and the head (*N* = 28, 27%). One hundred fifty-two of the participants (92%) reported that they had been given a pain-related medical diagnosis in relation to their pain. See Table 1 for a more detailed information.

**Table 1:** Sample characteristics (*N* = 165)

**Participant characteristics**	**Mean (SD, range) or *N* (%)**
Age, mean (SD, range)	43.68 (9.81, 18–68)
Self-identified sex at birth, *N* (%)	
Women	153 (93%)
Men	12 (7%)
Highest completed education level, *N* (%)	
Primary studies	13 (8%)
Secondary studies	94 (57%)
University studies	58 (35%)
Employment status, *N* (%)	
Students (in college)	7 (4%)
Employed	57 (35%)
Unemployed	54 (33%)
Retired	7 (4%)
Receiving a retirement pension	28 (17%)
Location of most frequent chronic pain, *N* (%)	
Head	28 (17%)
Cervical region	19 (12%)
Upper shoulder and upper limbs	30 (18%)
Thoracic region	2 (1%)
Abdominal region	3 (2%)
Lower back, lumbar spine, sacrum,	
and coccyx	60 (37%)
Lower limbs	21 (13%)
Pelvic region	2 (1%)
Course of chronic pain, *N* (%)	
Continuous	116 (70%)
Recurrent	49 (30%)
If recurrent, frequency chronic pain, *N* (%)	
Daily	27 (16%)
More than once a week	15 (9%)
Once a week	7 (4%)
Chronic pain duration in months, mean (SD)	90.46 (164.16)
Current diagnoses of chronic pain, *N* (%)	
Yes	152 (92%)
No	13 (8%)

### Procedures

The study protocol was approved by the Internal Review Board of the Universitat Rovira i Virgili. An online survey was designed to be completed using the Lime Survey software (https://www.limesurvey.org), with the data being saved on a secure server that is the property of the Universitat Rovira i Virgili. On the first page or screen of the survey, the information about the study purposes and instructions for participants were detailed. Before responding to the survey questions, participants had to express their consent to participate. The survey consisted of three sections, (1) demographic and descriptive information; (2) pain-related information; and (3) a series of questionnaires that assessed the variables used in this study, which are described below. A contact email address and a telephone number were provided to participants in case they required further assistance or need any help.

Participants were recruited from patients’ associations, support groups, and network discussion groups. Some participants also learned of the study by word of mouth. The authors contacted patient groups by email or through social networks and requested recruitment assistance by asking them to share information about the study with their members. Most of the groups contacted expressed a willingness to help with recruitment in this way.

### Measures

## Demographic Variables

Information about sex assigned at birth, age, highest completed education level, and employment status were collected for descriptive purposes.

## Pain-related Variables 

To ensure that participants had chronic pain, they were asked explicitly if they had pain anywhere in their body that lasted for over 3 months and, if so, to indicate the duration (in months) of this pain. They were also asked to describe the pain location(s), the location where they experienced pain most frequently, the time pattern of pain (e.g., constant, intermittent), and pain frequency. They were also asked whether or not they had a current pain diagnosis. Participants were then asked to rate their current pain intensity on a 0–10 numerical rating scale (NRS-11), where 0 = “no pain” and 10 = “worst pain imaginable.” A great deal of evidence supports the reliability and validity of 0–10 NRS measures for measuring pain intensity [40–43].

## Perceived Social Support

We used the Multidimensional Scale of Perceived Social Support (MSPSS) [44, 45] to assess perceived social support. This 12-item measure asks respondents to rate the extent to which they agree with statements about perceived social support from three sources: family, friends, and significant others. Respondents are asked to rate perceived social support using a 7-point Likert scale where 1 = “strongly disagree” and 7 = “strongly agree.” The responses are summed and divided by 12 to compute a total score that can range from 1 to 7, with higher scores indicating higher levels of perceived social support. The Spanish version of the MSPSS used here has been shown to be reliable and valid, supporting its use in the study’s population [46]. In the current study, the internal consistency coefficient (Cronbach’s alpha) of the total score was 0.93, indicating an excellent level of internal consistency reliability.

## Perceived Stress 

The 14-item perceived stress scale (PSS) developed by Cohen and colleagues [47] was used to measure the degree to which situations in one’s life are appraised as stressful. The PSS items reflect the experience of emotional stress. Sample items include “How often have you felt difficulties were piling up so high that you could not overcome them?” (positively scored) and “How often have you felt that things were going your way?” (negatively scored). Respondents are asked to rate the frequency of perceived stress related to each item during the last month using a 5-point scale (0 = “never” to 4 = “very often”). The total score can range from 0 to 56, with higher scores indicating higher levels of perceived stress. The Spanish version of the PSS used in this study has adequate psychometric properties, as evidenced by adequate levels of internal consistency, test–retest reliability, concurrent validity, and sensitivity [48]. In the study’s sample, the internal consistency (Cronbach’s alpha) of the PSS scores was 0.90, indicating excellent internal consistency reliability.

## Global Physical and Mental Health 

The PROMIS global mental health (GMH) and global physical health (GPH) scales [49] were used to assess participants’ global physical and mental health. This scale consists of ten items that provide general perceptions and evaluations of one’s physical and mental health. For the purposes of this study, we used only the eight items needed to calculate the global physical health score (composed by four items assessing overall physical health, physical function, pain, and fatigue) and the global mental health score (composed by four items assessing quality of life, mental health, satisfaction with social activities, and emotional problems). We then converted the summed scores of each scale to *T*-score values. We used the Spanish version provided in the PROMIS webpage (http://www.healthmeasures.net/explore-measurement-systems/promis). The Cronbach’s alpha coefficient of the GPH scale and the GMH scale for the current sample were 0.75 and 0.82, respectively, indicating adequate to good internal consistency for these measures.

### Data Analysis

We performed two hierarchical multiple linear regression analyses to test the study hypotheses. We first computed the means, standard deviations, skewness, and kurtosis of the study variables in order to ensure that they met the assumptions of the planned analysis. We computed Pearson correlations between the study variables and also evaluated multicollinearity by computing the variance inflation factor (VIF) and the tolerance values of the predictor variables.

In the first hierarchical multiple linear regression analysis, the criterion variable was the measure of global physical health, and in the second regression analysis, the criterion variable was the measure of global mental health. The predictor variables in both analyses were perceived social support and perceived stress. In the first step of both analyses, we entered two demographic variables (age and sex), pain intensity and chronic pain duration (in months), to control for their potentially confounding effects on both the predictor and criterion variables [50]. In step 2, we entered the primary study predictors as a block. In the third and final step, we tested the moderating effect of perceived social support on the association between perceived stress and the criterion variables by entering the perceived stress × perceived social support interaction term. Statistical analyses were performed using the Statistical Package for Social Sciences (SPSS) for Windows version 28.

## Results

### Preliminary Analyses

The analyses conducted to examine distributions of the predictor and criterion variables revealed that distributions were essentially normal for all variables. Table 2 shows the descriptive statistics for the study variables. As can be seen, the mean *T*-scores on the global mental health and global physical health scales were below the mean value of the standardized *T*-scores (i.e., mean value, 50). The average scores on the perceived stress measure in our sample (*N* = 165, mean = 31.19, SD = 10.23) are higher than those obtained in the Spanish validation sample [48] (*N* = 440, mean = 25, SD = 8.1; *t* = 7.76, *p* < 0.001), and the scores on the measure of perceived social support (*N* = 165, mean = 4.89, SD = 1.57) are lower than those obtained in the Spanish validation sample [51] (*N* = 991, mean = 5.2, SD = 1.24; *t* = 2.85, *p* < 0.01). In addition, a lack of multicollinearity was found as showed by (1) VIF values ranging from 1.03 to 1.35, which were all lower than 10 [52, 53], and (2) tolerance scores values being all very close to 1 (range = 0.740–0.973). Table 3 shows the Pearson correlations between the criteria and the predictor variables. As can be seen, coefficient values were all statistically significant.

**Table 2:** Descriptive statistics for the study variables

**Variable**	**Mean (SD)**	**Scale range**	**Skewness**	**Kurtosis**
Pain intensity (NRS-11)	6.53 (2.09)	0–10	−0.64	0.13
Perceived social support (MSPSS)	4.89 (1.57)	1–7	−0.47	−0.47
Perceived stress (PSS)	31.19 (10.23)	0–56	−0.14	−0.39
Physical health (PROMIS-GPH)	29.98 (7.00)	16.2–67.7	0.63	1.19
Mental health (PROMIS-GMH)	36.30 (9.17)	21.1–67.6	0.38	−0.15

NRS-11 = 0–10 numerical rating scale, *MSPSS* multidimensional scale of perceived social support, *PSS* perceived stress scale, *PROMIS-GPH* PROMIS global physical health, *PROMIS-GMH* PROMIS global mental health

**Table 3:** Pearson correlations between the criteria and the predictor variables

Variable	Perceived social support (MSPSS)	Perceived stress (PSS)
Perceived social support (MSPSS)	1.00	
Perceived stress (PSS)	−.47**	1.00
Global physical health (PROMIS-GPH)	.33**	−.50**
Global mental health (PROMIS-GMH)	.53**	−.77**

^**^p < 0.01

*MSPSS* multidimensional scale of perceived social support, *PSS* perceived stress scale, *PROMIS-GPH* PROMIS global physical health, *PROMIS-GMH* PROMIS global mental health

### Results of the Hierarchical Multiple Regression Analyses

#### Predicting Global Physical Health

Results of the hierarchical multiple regression analyses predicting global physical health are presented in Table 4. Age, sex, current pain intensity, and pain duration entered as control variables explained 32% of the variance in the criterion variable (*F*_(4,160)_ = 19.13, *p* < 0.001), which was explained primarily by pain intensity (*β* = −0.54, *t* = −7.59, *p* < 0.001). Perceived stress and perceived social support, entered as a block in step 2, explained an additional 16% of the variance (*F*_change (2,158)_ = 24.96, *p* < 0.001). However, only perceived stress made a statistically significant and independent contribution to the prediction of global physical health (*β* = −0.39, *t* = −5.94, *p* < 0.001). The perceived social support × perceived stress interaction was not statistically significant (*β* = 0.03, *t* = 0.55, *p* = 0.584).

**Table 4:** Summary of the hierarchical multiple regression analysis predicting global physical health

Step & variable	*R*^*2*^	*ΔR*^*2*^	*ΔF (p)*	*β*	*t* (*p*)	95% CI for* t*
1.Control variables	.32	.32	19.13 (< .001)			
Age				−.07	−.97 (.321)	−.15 to .05
Sex				.06	.92 (.361)	−1.89 to 5.16
Pain intensity				−.52	−7.59 (< .001)	−2.17 to −1.27
Chronic pain duration				−.11	−.69 (.094)	−.03 to .01
2. Predictor variables	.49	.16	24.96 (< .001)			
Social support				.05	.72 (.470)	−.37 to 0.80
Perceived stress				−.39	−5.94 (< .001)	−.36 to −.18
3. Interaction	.49	< .01	.30 (.584)			
Social support × perceived stress				.03	.55 (.584)	−.04 to .07

#### Predicting Global Mental Health

The results of the regression analyses predicting global mental health are presented in Table 5. As can be seen, the control variables (age, sex, current pain intensity, and pain duration) explained a 9% of the variance in the criterion variable (*F*_(4,160)_ = 4.29, *p* < 0.01), which was explained primarily by pain intensity (*β* = −0.23, *t* = −2.94, *p* < 0.01). Perceived social support and perceived stress explained an additional 65% of the variance (*F*_change (2,158)_ = 121.79, *p* < 0.001). Both perceived social support (*β* = 0.18, *t* = 3.31, *p* < 0.01) and perceived stress (*β* = −0.66, *t* = −12.09, *p* < 0.001) contributed independently to the prediction of global mental health. However, the interaction term was not statistically significant (*β* = −0.06, *t* = −1.27, *p* = 0.206).

**Table 5:** Summary of the hierarchical multiple regression analysis predicting global mental health

Step & variable	*R*^*2*^	*ΔR*^*2*^	*ΔF (p)*	*β*	*t* (*p*)	95% CI for *t*
1. Control variables	.09	.09	4.29 (.003)			
Age				−.06	−.79 (.431)	−.21 to .09
Sex				.05	.65 (.518)	−3.58 to 7.07
Pain intensity				−.23	−2.94 (.004)	−1.69 to −.33
Chronic pain duration				−.14	−1.84 (.068)	−.05 to 0.01
2.Predictor variables	.65	.55	121.79 (< .001)			
Perceived social support				.18	3.31 (.001)	.43 to 1.70
Perceived stress				−.66	−12.09 (< .001)	−.69 to −.49
3. Interaction	.63	.02	1.61 (.206)			
Social support × perceived stress				−.06	−1.27 (.206)	−.09 to .02

## Discussion

The findings from this study contribute to our understanding of the processes through which perceived social support and perceived stress may influence global physical and mental health by evaluating the two models that are used to explain these relationships [23, 24]. The results have important clinical and theoretical implications.

We found that perceived stress made an independent contribution to the prediction of both global physical and mental health, while perceived social support made an independent contribution only to the prediction of global mental health. In this study, the scores on the measures of perceived social support and global physical health were positive and significantly associated, but perceived social support was not an independent predictor of global physical health in the regression model. This finding is inconsistent with many, but not all, of the studies that have examined the effects of social support on physical health-related outcomes published over the recent decades [54]. The inconsistency in findings could potentially be due to the multidimensional and multifaceted nature of the social support construct. For example, in a recent study, it has been shown that different dimensions of social support (i.e., emotional, tangible or instrumental, interaction or exchange, and community support) have heterogeneous effects on individual physical and mental health [55].

The findings supported the direct-effects model, but not the stress-buffering model, in this sample of adults with chronic pain. In the current sample, the negative consequences of perceived stress on global physical and mental health were not buffered or mitigated by participants’ perceived social support. Previous research has found mixed evidence for the direct-effects model and for the stress-buffering model of social support in other samples of individuals with chronic pain. The findings here are consistent with previous research supporting the direct-effects model [33, 35]. The findings also support the possibility, but do not confirm this possibility given the cross-sectional nature of the data, that perceived social support and perceived stress may influence adjustment to chronic pain, consistent with a biopsychosocial model of chronic pain [9].

From a clinical perspective, and if future research supports a causal impact of perceived social support and perceived stress on global health and function in individuals with chronic pain, these findings reinforce the importance of including social support and stress management strategies when considering targets for pain interventions [56–58]. As indicated previously, the association between perceived stress and important health outcomes is well-established, including research supporting the effects of perceived stress on the onset and maintenance of chronic illness in general [59–61] and chronic pain in particular [14, 15]. Similarly, there is a great deal of research supporting associations between perceived social support and both physical and psychological outcomes [12]; some studies have also linked perceived social support with changes in physiological processes such as cardiovascular, neuroendocrine, and immune function [62]. Moreover, a lack of perceived social support has been linked to higher rates of morbidity and mortality in some studies [11, 63]. Promoting social support resources has been identified as a factor that can promote healthy behaviors such as adaptive coping strategies [64] and exercise practice [65], both of which are important to adjustment to chronic pain.

That said, it is also important to note that not all “social support” is healthy or beneficial. While we did not evaluate the effects of solicitous responses (i.e., a type of social response that involves encouraging patients with pain to engage in less activity or stop activity as a way to cope with pain, often intended as a supporting response), solicitous responses from spouses or partners have been consistently associated with poorer physical function, even if it is sometimes shown to be associated with better psychological function [51, 66, 67]. Thus, when encouraging individuals with chronic pain to seek and obtain more social support, it is important to educate them regarding the most useful types of social support — that is, unconditional support as opposed to conditional and pain-contingent support — so as to enhance long-term positive outcomes.

The lack of support for the stress-buffering model is consistent with some studies, cited previously, that also did not find moderating effects of perceived social support on the associations between perceived stress and pain-related outcomes. The findings here contribute to the literature on this topic, which could ultimately be used to help determine when buffering effects of perceived social support might and might not be most likely to be found. Factors that could potentially be associated with the presence of buffering effects could include the specific *measures* of perceived social support and perceived stress used. In our work, unlike some studies described in the introduction, perceived stress was assessed with a measure developed specifically for the purpose, the 14-item perceived stress scale (PSS) developed by Cohen and colleagues. Another factor that could potentially influence whether or not a stress-buffeting effect is identified is the specific *domains* of perceived social support and perceived stress assessed. A third factor is the participant’s personal characteristics, such as the type of the pain problem examined or the participant’s sex. In order to identify which of these personal and contextual factors impact the presence of a stress-buffering effect, multiple studies which assess different social support domains using different social support measures in samples with different characteristics are needed. The current analyses provide one such study for the research literature [23].

A number of the study’s limitations should be considered when interpreting the results of this study. First, the sample was one of convenience (i.e., individuals with chronic pain willing to complete an online survey) and was composed mostly of women. Although participants’ sex was controlled in the analyses, the findings might have been different if there had been a greater representation of men [68]. Additional research using other samples, including samples with more male participants or participants with other cultural backgrounds, is needed to help establish the reliability and generalizability of the results. Additional studies are also needed to examine how the nature of pain diagnosis and its severity could influence the results of the associations examined in this study. Unfortunately, participants in this study were asked only if they did or did not have a current pain diagnose: information about any diagnosis if there was one was not collected. Second, we collected data using an online survey, which did not allow us to verify the degree to which participants were genuine in their responses. However, participation was voluntary, and the participants were not compensated. Therefore, it seems highly unlikely that they provided misleading responses to the survey. Third, we used a cross-sectional design. While such a design is appropriate for testing the study hypotheses, we are not able to use the findings to evaluate the causal relationships among the study variables. Longitudinal research or clinical trials in which perceived social support or perceived stress are experimentally manipulated (e.g., by using random assignment to interventions which improve social connections and support and/or teach patients stress management strategies) are needed to evaluate the causal influences of changes in support or stress on subsequent function.

Despite the study’s limitations, the findings provide new important information regarding the role that both perceived social support and perceived stress play in the adjustment to chronic pain in adults. The potential importance of both factors was supported, although in this study only perceived stress was associated with both health domains examined. The findings also confirm the viability of the direct-effects model of social support and are consistent with the idea that perceived stress may have a direct and similar negative effect on health regardless of the amount of social support available. The inconsistent findings in the research literature concerning the stress-buffering model of social support suggests a need for research to help determine the conditions by which perceived social support might — or might not — buffer the negative impact of pain and stress on health in individuals with chronic pain. Ultimately, this body of research could provide an empirical basis for helping to determine which psychosocial factors to target in pain treatment — and perhaps when and for whom to target these factors — in order to maximize beneficial outcomes.

## References

[1] Goldberg DS, McGee SJ. Pain as a global public health priority. BMC Public Health. 2011;11:770. 21978149 10.1186/1471-2458-11-770 PMC3201926

[2] Reid KJ, Harker J, Bala MM, Truyers C, Kellen E, Bekkering GE, et al. Epidemiology of chronic non-cancer pain in Europe: narrative review of prevalence, pain treatments and pain impact. Curr Med Res Opin. 2011;27:449–62. 21194394 10.1185/03007995.2010.545813

[3] Breivik H, Collett B, Ventafridda V, Cohen R, Gallacher D. Survey of chronic pain in Europe: prevalence, impact on daily life, and treatment. Eur J Pain. 2006;10:287–333. 16095934 10.1016/j.ejpain.2005.06.009

[4] Dahlhamer J, Lucas J, Zelaya C, Nahin R, Mackey S, DeBar L, et al. Prevalence of chronic pain and high-impact chronic pain among adults — United States, 2016. MMWR Morb Mortal Wkly Rep. 2018;67:1001–6. 30212442 10.15585/mmwr.mm6736a2 PMC6146950

[5] Rikard SM, Strahan AE, Schmit KM, Guy GP. Chronic pain among adults — United States, 2019–2021. MMWR Morb Mortal Wkly Rep. 2023;72:379–85. 37053114 10.15585/mmwr.mm7215a1 PMC10121254

[6] Mills SEE, Nicolson KP, Smith BH. Chronic pain: a review of its epidemiology and associated factors in population-based studies. Br J Anaesth. 2019;123:e273–83. 31079836 10.1016/j.bja.2019.03.023 PMC6676152

[7] Wojcieszek A, Kurowska A, Majda A, Liszka H, Gądek A. The impact of chronic pain, stiffness and difficulties in performing daily activities on the quality of life of older patients with knee osteoarthritis. Int J Environ Res Public Health. 2022;19:16815. 36554695 10.3390/ijerph192416815 PMC9779661

[8] Gatchel RJ, Peng YB, Peters ML, Fuchs PN, Turk DC. The biopsychosocial approach to chronic pain: scientific advances and future directions. Psychol Bull. 2007;133:581–624. 17592957 10.1037/0033-2909.133.4.581

[9] Turk DC, Fillingim RB, Ohrbach R, Patel KV. Assessment of psychosocial and functional impact of chronic pain. J Pain. 2016;17:T21-49. 27586830 10.1016/j.jpain.2016.02.006

[10] Hulla R, Brecht D, Stephens J, Salas E, Jones C, Gatchel R. The biopsychosocial approach and considerations involved in chronic pain. Healthy Aging Res. 2019;08.

[11] Reblin M, Uchino BN. Social and emotional support and its implication for health. Curr Opin Psychiatry. 2008;21:201–5. 18332671 10.1097/YCO.0b013e3282f3ad89 PMC2729718

[12] Gruenewald TL, Seeman TE. Social support and physical health: links and mechanisms. Handbook of Behavioral Medicine. New York, NY: Springer New York; 2010. p. 225–36.

[13] Wiegner L, Hange D, Björkelund C, Ahlborg G. Prevalence of perceived stress and associations to symptoms of exhaustion, depression and anxiety in a working age population seeking primary care - an observational study. BMC Fam Pract. 2015;16. 10.1186/s12875-015-0252-7 PMC4377029 25880219

[14] White RS, Jiang J, Hall CB, Katz MJ, Zimmerman ME, Sliwinski M, et al. Higher perceived stress scale scores are associated with higher pain intensity and pain interference levels in older adults. J Am Geriatr Soc. 2014;62:2350–6. 25516031 10.1111/jgs.13135 PMC4362541

[15] Harris ML, Loxton D, Sibbritt DW, Byles JE. The influence of perceived stress on the onset of arthritis in women: findings from the Australian longitudinal study on women’s health. Ann Behav Med. 2013;46:9–18. 23436274 10.1007/s12160-013-9478-6

[16] Boring BL, Richter A, Mathur VA. Higher self-perceived stress reactivity is associated with increased chronic pain risk. Pain Rep. 2023;8. 10.1097/PR9.0000000000001068 PMC10036055 36969912

[17] Costa DC, Sá MJ, Calheiros JM. The effect of social support on the quality of life of patients with multiple sclerosis. Arq Neuropsiquiatr. 2012;70:108–13. 22311214 10.1590/s0004-282x2012000200007

[18] Benka J, Nagyova I, Rosenberger J, Calfova A, Macejova Z, Middel B, et al. Social support and psychological distress in rheumatoid arthritis: a 4-year prospective study. Disabil Rehabil. 2012;34:754–61. 22004369 10.3109/09638288.2011.619618

[19] Jensen MP, Moore MR, Bockow TB, Ehde DM, Engel JM. Psychosocial factors and adjustment to chronic pain in persons with physical disabilities: a systematic review. Arch Phys Med Rehabil. 2011:140–60. 10.1016/j.apmr.2010.09.021 PMC3028590 21187217

[20] Alphonsus KB, D’Arcy C. Is there an association between social support and pain among individuals living with multiple sclerosis? J Evid Based Integr Med. 2021;26. 10.1177/2515690X21991995 PMC7868479 33535805

[21] Khan A, Husain A. Social support as a moderator of positive psychological strengths and subjective well-being. Psychol Rep. 2010;106:534–8. 20524555 10.2466/pr0.106.2.534-538

[22] Brinker J, Cheruvu VK. Social and emotional support as a protective factor against current depression among individuals with adverse childhood experiences. Prev Med Rep. 2017;5:127–33. 27981026 10.1016/j.pmedr.2016.11.018 PMC5156603

[23] Cohen S, Wills TA. Stress, social support, and the buffering hypothesis. Psychol Bull. 1985;98:310–57. 3901065

[24] Solé E, Racine R, Tomé-Pires C, Galán S, Jensen MP, Miró J. Social factors, disability and depressive symptoms in adults with chronic pain. Clin J Pain. 2020;36(5):371–8. 32040011 10.1097/AJP.0000000000000815

[25] Bovier PA, Chamot E, Perneger TV. Perceived stress, internal resources, and social support as determinants of mental health among young adults. Qual Life Res. 2004;13:161–70. 15058797 10.1023/B:QURE.0000015288.43768.e4

[26] Szkody E, Stearns M, Stanhope L, McKinney C. Stress-buffering role of social support during COVID-19. Fam Process. 2021;60:1002–15. 33220082 10.1111/famp.12618 PMC7753728

[27] Raffaelli M, Andrade FCD, Wiley AR, Sanchez-Armass O, Edwards LL, Aradillas-Garcia C. Stress, social support, and depression: a test of the stress-buffering hypothesis in a Mexican sample. J Res Adolesc. 2013;23:283–9.

[28] Wang X, Cai L, Qian J, Peng J. Social support moderates stress effects on depression. Int J Ment Health Syst. 2014;8:41. 25422673 10.1186/1752-4458-8-41 PMC4242489

[29] Baek RN, Tanenbaum ML, Gonzalez JS. diabetes burden and diabetes distress: the buffering effect of social support. Ann Behav Med. 2014;48:145–55. 24550072 10.1007/s12160-013-9585-4 PMC4249652

[30] Carpenter KM, Fowler JM, Maxwell GL, Andersen BL. Direct and buffering effects of social support among gynecologic cancer survivors. Ann Behav Med. 2010;39:79–90. 20151235 10.1007/s12160-010-9160-1 PMC3645934

[31] Gellert P, Häusler A, Suhr R, Gholami M, Rapp M, Kuhlmey A, et al. Testing the stress-buffering hypothesis of social support in couples coping with early-stage dementia. PLoS ONE. 2018;13: e0189849. 29300741 10.1371/journal.pone.0189849 PMC5754077

[32] Kornblith AB, Herndon JE, Zuckerman E, Viscoli CM, Horwitz RI, Cooper MR, et al. Social support as a buffer to the psychological impact of stressful life events in women with breast cancer. Cancer. 2001;91:443–54. 11180093 10.1002/1097-0142(20010115)91:2<443::aid-cncr1020>3.0.co;2-z

[33] Miró J, de la Vega R, Gertz K, Jensen MP, Engel JM. The role of perceived family social suport and parental solicitous responses in adjustment to bothersome pain in young people with disabilities. Disabil Rehabil. 2019;41(6):641–8. 29130816 10.1080/09638288.2017.1400594 PMC6289892

[34] De la Vega R, Molton IR, Miró J, Smith AE, Jensen MP. Changes in perceived social support predict changes in depressive symptoms in adults with physical disability. Disabil Health J. 2019;12(2):214–9. 30314820 10.1016/j.dhjo.2018.09.005

[35] Jensen MP, Smith AE, Bombardier CH, Yorkston KM, Miró J, Molton IR. Social support, depression, and physical disability: age and diagnostic group effects. Disabil Health J. 2013;7(2):164–72. 24680045 10.1016/j.dhjo.2013.11.001

[36] Brandstetter S, Riedelbeck G, Steinmann M, Ehrenstein B, Loss J, Apfelbacher C. Pain, social support and depressive symptoms in patients with rheumatoid arthritis: testing the stress-buffering hypothesis. Rheumatol Int. 2017;37:931–6. 28124095 10.1007/s00296-017-3651-3

[37] Penninx BW, van Tilburg T, Deeg DJ, Kriegsman DM, Boeke AJ, van Eijk JT. Direct and buffer effects of social support and personal coping resources in individuals with arthritis. Soc Sci Med. 1997;44:393–402. 9004373 10.1016/s0277-9536(96)00156-6

[38] Affleck G, Pfeiffer C, Tennen H, Fifield J. Social support and psychosocial adjustment to rheumatoid arthritis quantitative and qualitative findings. Arthritis Care Res. 1988;1:71–7.

[39] Curtis R, Groarke A, Coughlan R, Gsel A. The influence of disease severity, perceived stress, social support and coping in patients with chronic illness: A 1 year follow up. Psychol Health Med. 2004;9:456–75.

[40] Jensen MP, Karoly P. Self-report scales and procedures for assessing pain in adults. In: Turk DC, Melzack R, editors. Handbook of pain assessment. 3rd ed. New York: The Guilford Press; 2011. p. 19–44.

[41] Safikhani S, Gries KS, Trudeau JJ, Reasner D, Rüdell K, Coons SJ, et al. Response scale selection in adult pain measures: results from a literature review. J Patient Rep Outcomes. 2018. 10.1186/s41687-018-0053-6 PMC6127068 30238085

[42] Hjermstad MJ, Fayers PM, Haugen DF, Caraceni A, Hanks GW, Loge JH, et al. Studies comparing numerical rating scales, verbal rating scales, and visual analogue scales for assessment of pain intensity in adults: a systematic literature review. J Pain Symptom Manage. 2011:1073–93. 10.1016/j.jpainsymman.2010.08.016 21621130

[43] Karcioglu O, Topacoglu H, Dikme O, Dikme O. A systematic review of the pain scales in adults: which to use? Am J Emerg Med. 2018:707–14. 10.1016/j.ajem.2018.01.008 29321111

[44] Zimet GD, Powell SS, Farley GK, Werkman S, Berkoff KA. Psychometric characteristics of the multidimensional scale of perceived social support. J Pers Assess. 1990;55:610–7. 2280326 10.1080/00223891.1990.9674095

[45] Zimet GD, Dahlem NW, Zimet SG, Farley GK. The multidimensional scale of perceived social support. J Pers Assess. 1988;52:30–41. 10.1080/00223891.1990.9674095 2280326

[46] López Y, Fernández J, Navarro-Pardo E, Murphy M. Confirmatory factor analysis for the multidimensional scale of perceived social support in a sample of early retirees enrolled in university programs. Clin Gerontol. 2016;14:1–12. 10.1080/07317115.2016.1199077 28452640

[47] Cohen S, Kamarck T, Mermelstein R. A global measure of perceived stress. J Health Soc Behav. 1983;24:385–96. 6668417

[48] Remor E. Psychometric properties of a European Spanish version of the perceived stress scale (PSS). Span J Psychol. 2006;9:86–93. 16673626 10.1017/s1138741600006004

[49] Hays RD, Bjorner JB, Revicki DA, Spritzer KL, Cella D. Development of physical and mental health summary scores from the patient-reported outcomes measurement information system (PROMIS) global items. Qual Life Res. 2009;18:873–80. 19543809 10.1007/s11136-009-9496-9 PMC2724630

[50] Fillingim RB, King CD, Ribeiro-Dasilva MC, Rahim-Williams B, Riley JL. Sex, gender, and pain: a review of recent clinical and experimental findings. J Pain. 2009:447–85. 10.1016/j.jpain.2008.12.001 PMC2677686 19411059

[51] López-Martínez AE, Esteve-Zarazaga R, Ramírez-Maestre C. Perceived social support and coping responses are independent variables explaining pain adjustment among chronic pain patients. J Pain. 2008;9:373–9. 18203665 10.1016/j.jpain.2007.12.002

[52] Bowerman BL, O’Connell RT. Linear statistical models : an applied approach. 2nd ed. Boston: PWS-Kent Pub. Co; 1990.

[53] Myers RH. Classical and modern regression with applications. 2nd ed. Boston: Duxbury Press; 1990.

[54] Racine M, Galán S, de la Vega R, Tomé-Pires C, Solé E, Nielson WR, Miró J, Moulin DE, Jensen MP. Pain-related activity management patterns and function in patients with Fibromyalgia Syndrome. Clin J Pain. 2018;34(2):122–9. 28591081 10.1097/AJP.0000000000000526

[55] Yang F, Jiang Y. Heterogeneous influences of social support on physical and mental health: evidence from China. Int J Environ Res Public Health. 2020;17. 10.3390/ijerph17186838 PMC7558190 32962140

[56] Finlay KA, Peacock S, Elander J. Developing successful social support: an interpretative phenomenological analysis of mechanisms and processes in a chronic pain support group. Psychol Health. 2018;33:846–71. 29300123 10.1080/08870446.2017.1421188

[57] Vambheim SM, Kyllo TM, Hegland S, Bystad M. Relaxation techniques as an intervention for chronic pain: a systematic review of randomized controlled trials. Heliyon. 2021;7: e07837. 34485731 10.1016/j.heliyon.2021.e07837 PMC8405991

[58] Davis MC, Zautra AJ, Wolf LD, Tennen H, Yeung EW. Mindfulness and cognitive-behavioral interventions for chronic pain: differential effects on daily pain reactivity and stress reactivity. J Consult Clin Psychol. 2015;83:24–35. 25365778 10.1037/a0038200 PMC4323633

[59] Harris ML, Oldmeadow C, Hure A, Luu J, Loxton D, Attia J. Stress increases the risk of type 2 diabetes onset in women: a 12-year longitudinal study using causal modelling. Samocha-Bonet D, editor. PLoS One. 2017;12:e0172126. 10.1371/journal.pone.0172126 PMC5319684 28222165

[60] Salleh MR. Life event, stress and illness. Malays J Med Sci. 2008;15:9–18. 22589633 PMC3341916

[61] Renzaho AMN, Houng B, Oldroyd J, Nicholson JM, D’Esposito F, Oldenburg B. Stressful life events and the onset of chronic diseases among Australian adults: findings from a longitudinal survey. Eur J Public Health. 2013;24:57–62. 23397581 10.1093/eurpub/ckt007

[62] Uchino BN. Social support and health: a review of physiological processes potentially underlying links to disease outcomes. J Behav Med. 2006;29:377–87. 16758315 10.1007/s10865-006-9056-5

[63] Holt-Lunstad J, Smith TB, Baker M, Harris T, Stephenson D. Loneliness and social isolation as risk factors for mortality. Perspect Psychol Sci. 2015;10:227–37. 25910392 10.1177/1745691614568352

[64] Holtzman S, Newth S, Delongis A. The role of social support in coping with daily pain among patients with rheumatoid arthritis. J Health Psychol. 2004;9:677–95. 15310421 10.1177/1359105304045381

[65] Casado BL, Resnick B, Zimmerman S, Nahm E-S, Orwig D, Macmillan K, et al. Social support for exercise by experts in older women post-hip fracture. J Women Aging. 2009;21:48–62. 19199153 10.1080/08952840802633719 PMC2791694

[66] McCracken LM. Social context and acceptance of chronic pain: the role of solicitous and punishing responses. Pain. 2005;113:155–9. 15621376 10.1016/j.pain.2004.10.004

[67] Miró J, Huguet A, Jensen MP. Pain beliefs predict pain intensity and pain status in children: uselfulness of the pediatric version of the survey of pain attitudes. Pain Med. 2014;15:887–97. 24393548 10.1111/pme.12316

[68] Landman-Peeters KMC, Hartman CA, van der Pompe G, den Boer JA, Minderaa RB, Ormel J. Gender differences in the relation between social support, problems in parent-offspring communication, and depression and anxiety. Soc Sci Med. 2005;60:2549–59. 15814180 10.1016/j.socscimed.2004.10.024

